# Supplementary material for: Quantifying the availability of seasonal surface water and identifying the drivers of change within tropical forests in Cambodia
Source: PLoS One. 2024 Jul 29;19(7):e0307964. doi: 10.1371/journal.pone.0307964 (PMC11285917; doi:10.1371/journal.pone.0307964)
Supplement: S1 Fig — This map shows the location of the Economic Land Concessions (ELCs, highlighted in red) present within the Study Site which is the giant ibis estimated distribution (yellow envelope). The protected areas (PAs) are also shown (grey) to give spatial context of the ELCs in relation to Cambodia’s PAs. Protected areas, Economic Land Concessions and water bodies data included in this figure has been published by Open Development Cambodia herein are licensed under a CC BY-SA 4.0. The giant ibis distribution data included in this figure has been reprinted from [34] under a CC BY licence, with permission from BirdLife International, original copyright [2019]. (DOCX) [file pone.0307964.s001.docx]

**S1 Fig. Map of Economic Land Concessions within the study area.**


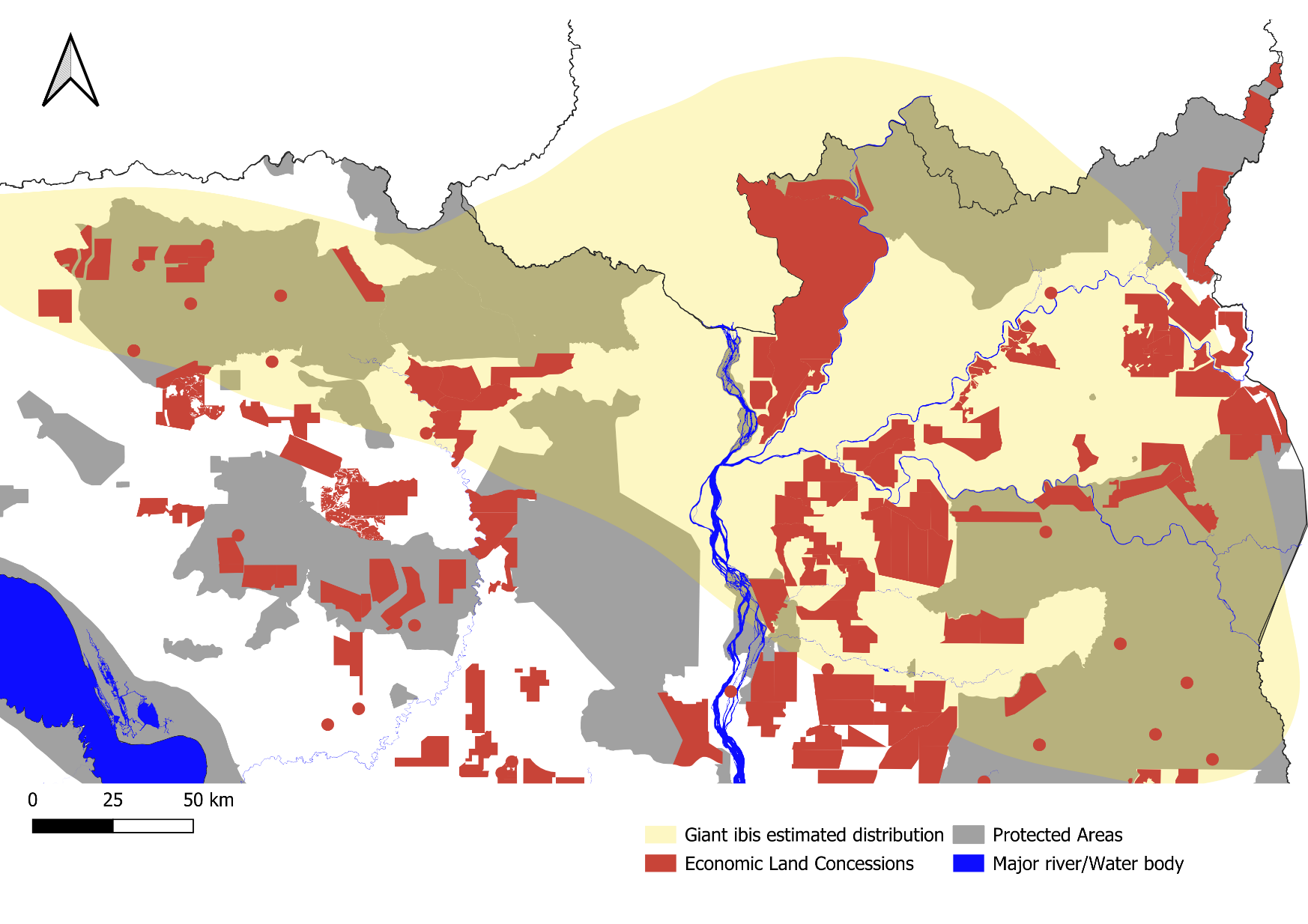
This map shows the location of the Economic Land Concessions (ELCs, highlighted in red) present within the Study Site which is the giant ibis estimated distribution (yellow envelope). The protected areas (PAs) are also shown (grey) to give spatial context of the ELCs in relation to Cambodia’s PAs. Protected areas, Economic Land Concessions and water bodies data included in this figure has been published by Open Development Cambodia herein are licensed under a CC BY-SA 4.0. The giant ibis distribution data included in this figure has been reprinted from [1] under a CC BY licence, with permission from BirdLife International, original copyright [2019].

**References**

1. Birdlife International. Thaumatibis gigantea. The IUCN Red List of Threatened Species 2018. IUCN; 2018.
