# Supplementary material for: Quantifying the availability of seasonal surface water and identifying the drivers of change within tropical forests in Cambodia
Source: PLoS One. 2024 Jul 29;19(7):e0307964. doi: 10.1371/journal.pone.0307964 (PMC11285917; doi:10.1371/journal.pone.0307964)
Supplement: S2 Fig — This map shows the location and distribution of the 438 giant ibis nests (black triangles), recorded between 2003 and 2020 within the northern plains protected areas: A) Kulen Promtep Wildlife Sanctuary, B) Prey Preah Roka Wildlife Sanctuary and C) Chhaeb Wildlife Sanctuary. The nest location data has been plotted over the surface water transition map to show changes in surface water between 2000–2004 and 2016–2020. (DOCX) [file pone.0307964.s002.docx]

**S4 Fig. Map to show the location of giant ibis nest sites.**

This map shows the location and distribution of the 438 giant ibis nests (black triangles), recorded between 2003 and 2020 within the northern plains protected areas: A) Kulen Promtep Wildlife Sanctuary, B) Prey Preah Roka Wildlife Sanctuary and C) Chhaeb Wildlife Sanctuary. The nest location data has been plotted over the surface water transition map to show changes in surface water between 2000-2004 and 2016-2020. Country boundary data is reprinted from HDX under a Creative Commons Attribution 4.0 International licence. Protected areas and water bodies data included in this figure has been published by Open Development Cambodia herein are licensed under a CC BY-SA 4.0. The giant ibis distribution data included in this figure has been reprinted from [1] under a CC BY licence, with permission from BirdLife International, original copyright [2019].


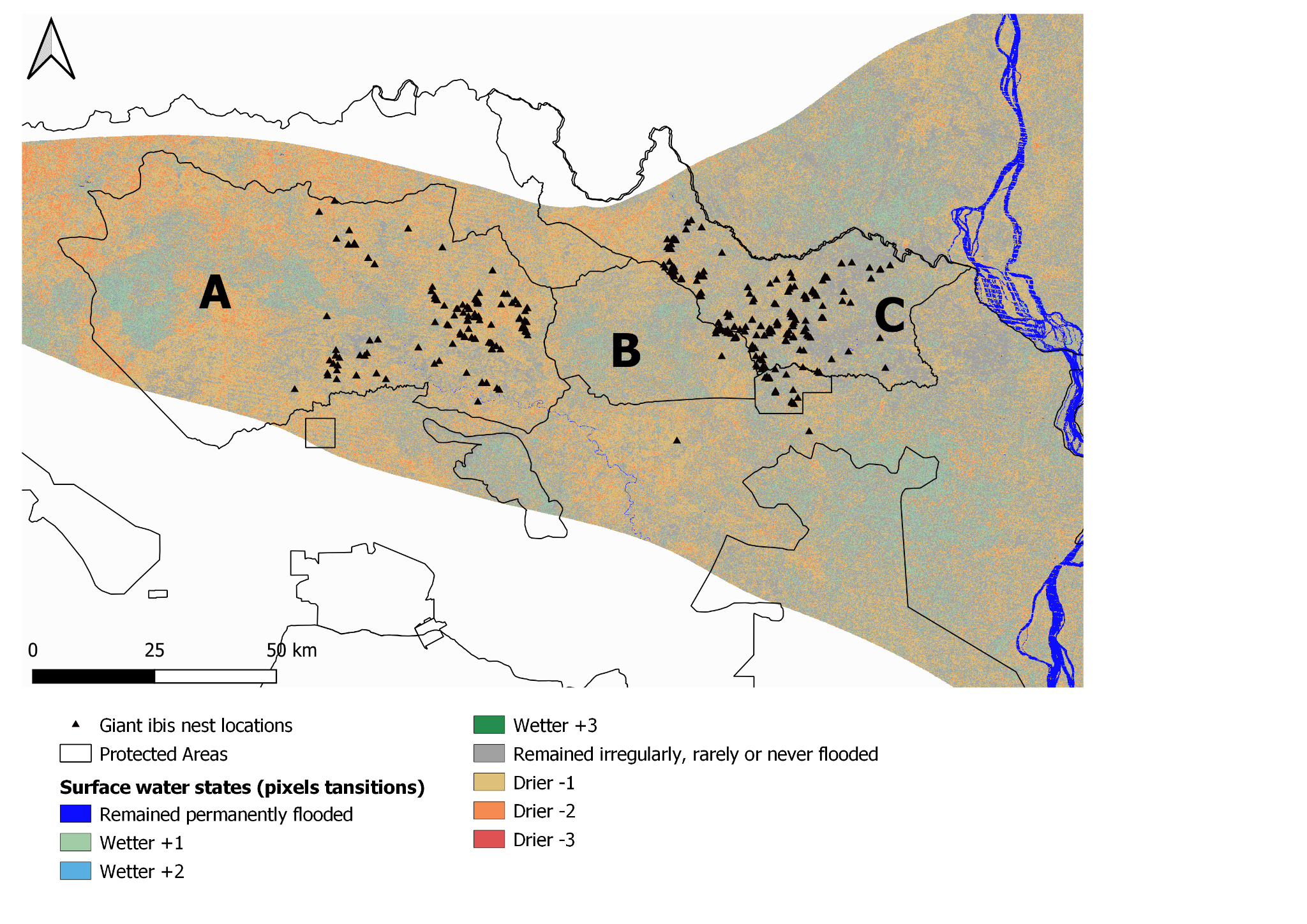


**References**

1. Birdlife International. Thaumatibis gigantea. The IUCN Red List of Threatened Species 2018. IUCN; 2018.
