# Supplementary material for: Quantifying the availability of seasonal surface water and identifying the drivers of change within tropical forests in Cambodia
Source: PLoS One. 2024 Jul 29;19(7):e0307964. doi: 10.1371/journal.pone.0307964 (PMC11285917; doi:10.1371/journal.pone.0307964)

**S7 Fig. Figure to show trend analysis results for mean annual precipitation.**

This figure shows how the mean annual precipitation across the study site changes over time. The data is from the CHIRPS database accessed from Google Earth Engine and plotted between 2000 and 2020. We plotted a linear model trendline onto the plot to look at the overall changes. This figure shows a decline in the mean annual precipitation over time.


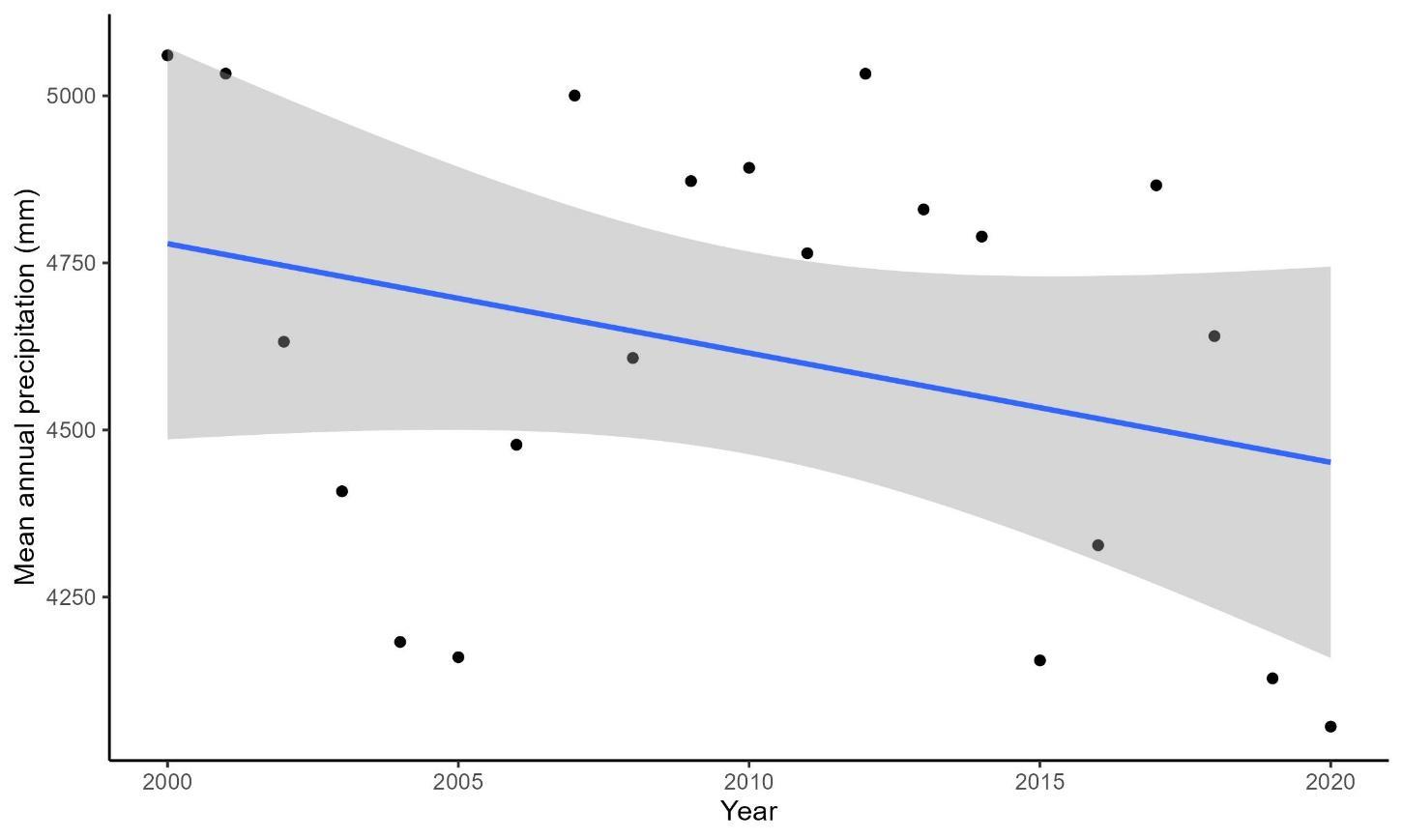

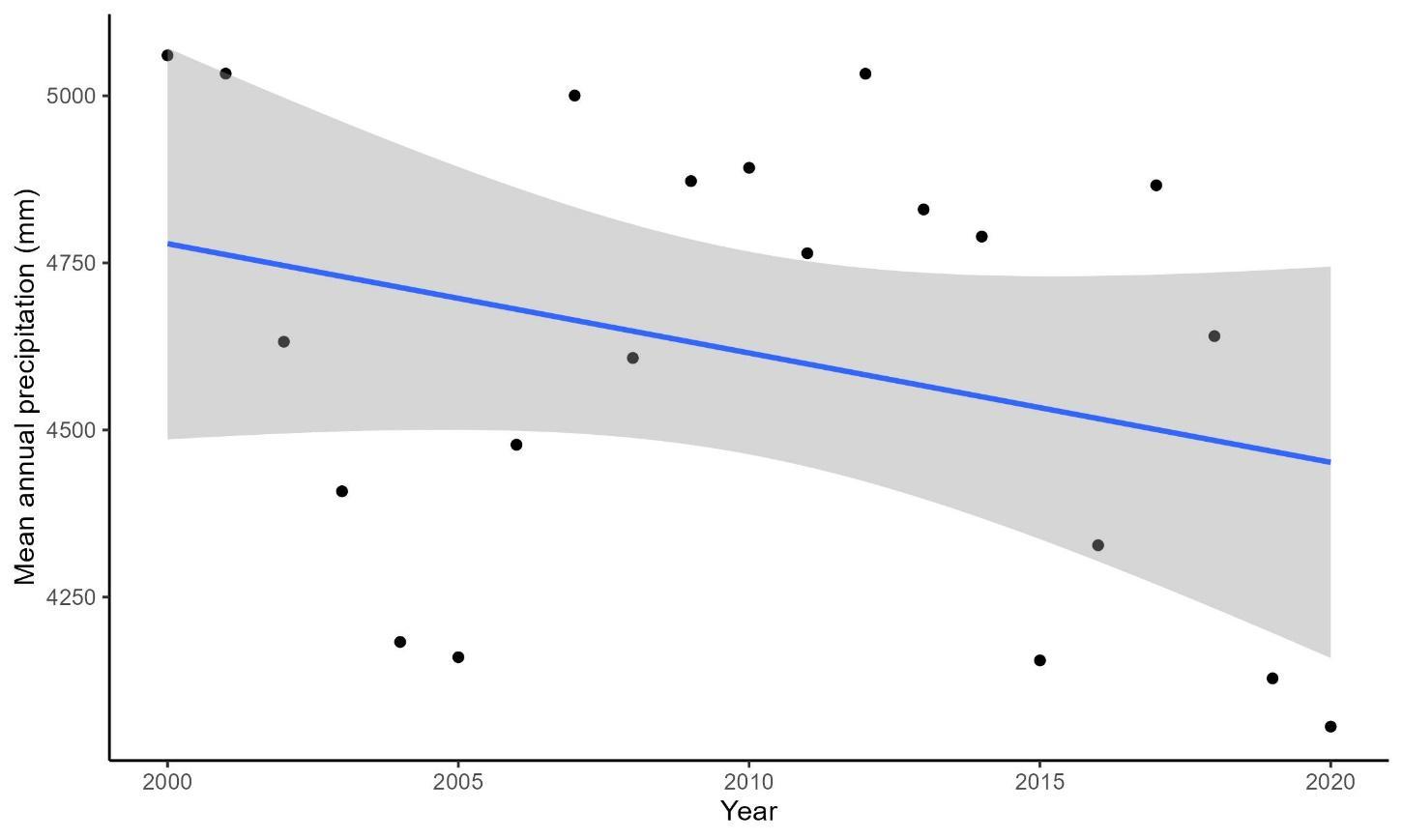

Supplement: S3 Fig — (DOCX) [file pone.0307964.s003.docx]
