# Supplementary material for: Quantifying the availability of seasonal surface water and identifying the drivers of change within tropical forests in Cambodia
Source: PLoS One. 2024 Jul 29;19(7):e0307964. doi: 10.1371/journal.pone.0307964 (PMC11285917; doi:10.1371/journal.pone.0307964)
Supplement: S1 Text — (DOCX) [file pone.0307964.s004.docx]

**S3 Text. Additional information on the covariate data used within the analysis.**

As part of the analysis we tested the influence of precipitation on availability of surface water. We extracted precipitation data from Climate Hazards Group InfraRed Precipitation With Station Data (CHIRPS) which is a database of rainfall data that began in 1981, combining 5km (0.05°) resolution satellite imagery with in-situ station data to create a gridded rainfall time series [1]. We also included data on the topography of the study site and how it affected surface water availability from STRM [2].

We compiled all the covariate raster data together into a uniform raster stack. We then rasterized all the covariate vectors and surface water data to a 500m resolution to match the resolution of the topography layer. We created a binary system for the land use polygons designating pixels within Economic Land Concessions (ELC) or Protected Areas (PA) [3]. Pixels were valued as one if they were within an ELC or PA and zero for an area outside either of those land use types. A pixel was given the value of one if the land use type covered the centre of the pixel using the *Rasterize* function in the computer program R [4]. The *Rasterize* function was again used to denote a pixel with or without roads and for this process all pixels touched by a road line were classed as one and those that did not were classed as zero [4]. We also calculated the distance from each pixel to the nearest road. All covariates were scaled to a mean of zero and a standard deviation of one before analysis.

**References**

1. Funk C, Peterson P, James LMPD, Shraddhanand S, Husak G, Rowland J, et al. The climate hazards infrared precipitation with stations—a new environmental record for monitoring extremes. 2015. doi:10.1038/sdata.2015.66

2. NASA JPL. NASADEM Merged DEM Global 1 arc second V001. NASA EOSDIS Land Processes DAAC. 2020. doi:10.5067/MEaSUREs/NASADEM/NASADEM_HGT.001

3. Open Development Cambodia. Open Development Cambodia, Map Catalogue. 2017. Available: https://opendevelopmentcambodia.net/layers

4. Perpiñán O, Hijmans R. rasterVis. 2023. Available: https://oscarperpinan.github.io/rastervis/
