# Supplementary material for: Quantifying the availability of seasonal surface water and identifying the drivers of change within tropical forests in Cambodia
Source: PLoS One. 2024 Jul 29;19(7):e0307964. doi: 10.1371/journal.pone.0307964 (PMC11285917; doi:10.1371/journal.pone.0307964)
Supplement: S2 Text — (DOCX) [file pone.0307964.s005.docx]

**S5 Text. Field methodology for giant ibis nest monitoring.**

Giant ibis nests are monitored as part of the wider Bird Nest Protection Programme run in the northern plains of Cambodia by the Wildlife Conservation Society (WCS) and the Ministry of Environment which has been running in the Northern Plains since 2002 [1,2]. The main way that giant ibis nests are located is through active nest searching within the protected areas which is undertaken by the USAID GPL and WCS biodiversity teams [3]. This data was collected under the permissions of the collaboration and access between WCS and the Ministry of Environment. This data collection begins around May each year in time for the giant ibis breeding season. Giant ibis breeding pairs usually return to the same nesting sites year on year so these trees as well as the wider landscape and new potential nesting areas are checked annually. This is conducted via specific biodiversity surveying and monitoring activities, as part of the Spatial Monitoring and Reporting Tool (SMART) data collection scheme or via patrols within the Protected Areas or Community Protected Areas [3]. The SMART data collected gives information on the levels of effort employed for nest searching as well as specific spatial data on illegal activities and other species seen whilst on patrol [3].

Nest searching is also conducted by local people that live within or close to the protected areas [1]. The nests are located and reported to WCS by community members, often community protected area rangers and nest protectors who have been selected and employed by WCS [1]. Once a nest is located a nest guardian is assigned to monitor the nest until the chicks have fledged [1]. All nest guardians are given annual contracts and payment specifically to find and monitor nests until the chicks have fledged successfully [1] which is between September November for the giant ibis [2]. They remain ~150m from the nest and do not create any disturbance themselves to the nesting birds. They also must follow the rules of the protected area by refraining from hunting or logging within the protected area [1]. The nest guardians themselves often live or work close by to the site and monitor the nest daily keeping track of any developments within the nest or any illegal activity in the surrounding area that they see [1,3]. They are trained by WCS staff to collect appropriate data on the location of the nest, habitat type, number of eggs, chicks, any disturbance or information if the nest fails [5]. The nest guardians check in once a week with WCS community wildlife rangers and monthly with WCS monitoring staff to check on their progress [1,2].

**References**

1. Harrison S, Mao K. Bird Nest Protection Programme in the Northern Plains of Cambodia 2009-2017. Wildlife Conservation Society Cambodia; 2017.

2. Clements T, Garrett L, Johna A, Keo O, Sreng K, Bunnat P, et al. Case study: Bird Nest Protection Program in the Northern Plains of Cambodia. WCS TransLinks Program; 2009.

3. USAID GPL. Draft Guidelines and Payment Policy for Bird Nest Protection Program in Prey Lang Extended Landscape. USAID GPL ;
