# Supplementary material for: Quantifying the availability of seasonal surface water and identifying the drivers of change within tropical forests in Cambodia
Source: PLoS One. 2024 Jul 29;19(7):e0307964. doi: 10.1371/journal.pone.0307964 (PMC11285917; doi:10.1371/journal.pone.0307964)
Supplement: S1 Table — The table below summarises the 16 transition categories that we used to define the pixel transitions. The transitions categories for the first (2000–2004) and last periods (2016–2020) were defined. The transition value denotes the number of categories that the pixel has changed by between the two periods. QGIS was used to calculate the percentage area of each surface water state and transition category to reflect the extent of the change. (DOCX) [file pone.0307964.s006.docx]

### **S2 Table. Table of surface water transition categories.**

The table below summarises the 16 transition categories that we used to define the pixel transitions. The transitions categories for the first (2000-2004) and last periods (2016-2020) were defined. The transition value denotes the number of categories that the pixel has changed by between the two periods. QGIS was used to calculate the percentage area of each surface water state and transition category to reflect the extent of the change.

| **Transition category** | **Initial state (2000-2004)** | **End state (2016-2020)** | **Transition value** |
| --- | --- | --- | --- |
| 1 | Permanently flooded | Permanently flooded | 0 |
| 2 | Irregularly flooded | Permanently flooded | +1 |
| 3 | Rarely flooded | Permanently flooded | +2 |
| 4 | Never flooded | Permanently flooded | +3 |
| 5 | Permanently flooded | Irregularly flooded | -1 |
| 6 | Irregularly flooded | Irregularly flooded | 0 |
| 7 | Rarely flooded | Irregularly flooded | +1 |
| 8 | Never flooded | Irregularly flooded | +2 |
| 9 | Permanently flooded | Rarely flooded | -2 |
| 10 | Irregularly flooded | Rarely flooded | -1 |
| 11 | Rarely flooded | Rarely flooded | 0 |
| 12 | Never flooded | Rarely flooded | +1 |
| 13 | Permanently flooded | Never flooded | -3 |
| 14 | Irregularly flooded | Never flooded | -2 |
| 15 | Rarely flooded | Never flooded | -1 |
| 16 | Never flooded | Never flooded | 0 |
