# Supplementary material for: Quantifying the availability of seasonal surface water and identifying the drivers of change within tropical forests in Cambodia
Source: PLoS One. 2024 Jul 29;19(7):e0307964. doi: 10.1371/journal.pone.0307964 (PMC11285917; doi:10.1371/journal.pone.0307964)
Supplement: S2 Table — This table summarises the percentile value comparison carried out for sensitivity checking for the giant ibis suitable habitat area. We calculated the 65th, 75th and 85th percentiles of the data for comparison. We chose the 75th percentile to define the area that we considered as suitable habitat for the giant ibis. There was little difference between the 65th and 85th percentiles when compared with the 75th percentile values so the 75th was selected. We used this value to determine the area of suitable giant ibis habitat lost between the two time periods in both the wet and dry season. (DOCX) [file pone.0307964.s007.docx]

**S6 Table. Table to summarise the sensitivity checking results for the giant ibis habitat availability analysis.**

This table summarises the percentile value comparison carried out for sensitivity checking for the giant ibis suitable habitat area. We calculated the 65th, 75th and 85th percentiles of the data for comparison. We chose the 75th percentile to define the area that we considered as suitable habitat for the giant ibis. There was little difference between the 65th and 85th percentiles when compared with the 75th percentile values so the 75th was selected. We used this value to determine the area of suitable giant ibis habitat lost between the two time periods in both the wet and dry season.

|  | **2000-2004** | | **2016-2020** | |
| --- | --- | --- | --- | --- |
| **Percentile** | **Dry season median (**$\boldsymbol{km}^{2}$**)** | **Wet season median (**$\boldsymbol{km}^{2}$**)** | **Dry season median (**$\boldsymbol{km}^{2}$**)** | **Wet season median (**$\boldsymbol{km}^{2}$**)** |
| 65th | 6150 | 8351 | 4145 | 6632 |
| 75th | 7050 | 8351 | 5259 | 6633 |
| 85th | 8470 | 10180 | 7202 | 9495 |
