# Supplementary material for: Quantifying the availability of seasonal surface water and identifying the drivers of change within tropical forests in Cambodia
Source: PLoS One. 2024 Jul 29;19(7):e0307964. doi: 10.1371/journal.pone.0307964 (PMC11285917; doi:10.1371/journal.pone.0307964)
Supplement: S3 Table — Table to summarise the linear model results conducted for the mean annual precipitation over time. We created a linear model to explore the trend in precipitation over time. (DOCX) [file pone.0307964.s008.docx]

**S8 Table. Table to show trend analysis results for mean annual precipitation.**

Table to summarise the linear model results conducted for the mean annual precipitation over time. We created a linear model to explore the trend in precipitation over time and the model and results are shown below. The model equation is included below:

*lm(precip ~ year)*

| **Residuals** | **Min** | **1Q** | **Median** | **3Q** | **Max** |
| --- | --- | --- | --- | --- | --- |
|  | -536.9 | -321.4 | 156.1 | 0.14 | 450.6 |

|  | **Estimate** | **Standard error** | **t-value** | **Pr(>\|t\|)** |
| --- | --- | --- | --- | --- |
| Intercept | 37490.38 | 24055.71 | 1.56 | 0.14 |
| year | -16.35 | 11.97 | -1.37 | 0.19 |

| **Residual standard error: 332.1 on 19 degrees of freedom** | | | |
| --- | --- | --- | --- |
| **Multiple R-squared** | **Adjusted R-squared** | **F-stat** | **p-value** |
| 0.0895 | 0.04 | 1.868 | 0.19 |
