# Supplementary material for: Quantifying the availability of seasonal surface water and identifying the drivers of change within tropical forests in Cambodia
Source: PLoS One. 2024 Jul 29;19(7):e0307964. doi: 10.1371/journal.pone.0307964 (PMC11285917; doi:10.1371/journal.pone.0307964)
Supplement: S4 Table — Table to summarise the Pearson correlation test results for the relationship between surface water availability and precipitation and dry water availability during the dry season and precipitation. We found no correlation for either of the tests. (DOCX) [file pone.0307964.s009.docx]

**S9 Table. Table to summarise the Pearsons correlation test results**

Table to summarise the Pearson correlation test results for the relationship between surface water availability and precipitation and surface water availability during the dry season and precipitation. We found no correlation for either of the tests.

|  | **t** | **df** | **p-value** | **95% confidence interval** | **Sample estimates: cor** |
| --- | --- | --- | --- | --- | --- |
| Surface water ~ precipitation | -0.69 | 19 | 0.5014 | -05501254, – 0.2962236 | -0.1553256 |
| Dry season surface water ~ precipitation | -1.81 | 19 | 0.0859 | -0.69956115, – 0.05743205 | -0.3837694 |
