# Supplementary material for: Quantifying the availability of seasonal surface water and identifying the drivers of change within tropical forests in Cambodia
Source: PLoS One. 2024 Jul 29;19(7):e0307964. doi: 10.1371/journal.pone.0307964 (PMC11285917; doi:10.1371/journal.pone.0307964)
Supplement: S5 Table — These tables summarise the results from A) Model 1 which tested for general changes (drying and flooding) across the study site; B) Model 2 which tested for the probability of extreme drying events which are defined as those pixels that transitioned by two or more categories drier; and C) Model 3 which tested for the probability of extreme flooding events which are defined as those pixels that transitioned by two or more categories wetter. It displays the effect of the selected covariates on the surface water within the study site using the gaussian general model. Here we have shown the mean values, the upper and lower quantile ranges. The bold values identify those covariates that have a significant effect on the surface water (have ranges that do not overlap zero). (DOCX) [file pone.0307964.s010.docx]

**S10 Table. Tables of results from the INLA Models**.

These tables summarise the results from A) Model 1 which tested for general changes (drying and flooding) across the study site; B) Model 2 which tested for the probability of extreme drying events which are defined as those pixels that transitioned by two or more categories drier; and C) Model 3 which tested for the probability of extreme flooding events which are defined as those pixels that transitioned by two or more categories wetter. It displays the effect of the selected covariates on the surface water within the study site using the gaussian general model. Here we have shown the mean values, the upper and lower quantile ranges. The bold values identify those covariates that have a significant effect on the surface water (have ranges that do not overlap zero).

| A) | | |
| --- | --- | --- |
| Covariates | Mean | Range (0.025 – 0.975) |
| **Intercept** | **-0.17** | **(-0.18 - -0.16)** |
| **Roads** | **0.02** | **(0.02 – 0.03)** |
| **Elevation** | **0.05** | **(0.04 - 0.06)** |
| **Economic Land Concessions** | **-0.04** | **(-0.05 - -0.02)** |
| **Protected Areas** | **-0.05** | **(-0.06 - -0.02)** |

| B) | | |
| --- | --- | --- |
| Covariates | Mean | Range (0.025 – 0.975) |
| **Intercept** | **-6.82** | **(-7.35 - -6.30)** |
| **Roads** | **-0.30** | **(-0.35 - -0.24)** |
| **Elevation** | **0.33** | **(0.04 – 0.59)** |
| **Economic Land Concessions** | **0.34** | **(0.03 – 0.64)** |
| **Protected Areas** | **-0.58** | **(-0.91 - -0.26)** |

| C) | | |
| --- | --- | --- |
| Covariates | Mean | Range (0.025 – 0.975) |
| **Intercept** | **-9.25** | **(-9.82 - -8.70)** |
| Roads | -0.09 | (-0.25 – 0.01) |
| Elevation | 0.01 | (-0.33 – 0.34) |
| Economic Land Concessions | -0.20 | (-0.87 – 0.43) |
| **Protected Areas** | **-1.16** | **(-2.09 - -0.29)** |
