# Supplementary material for: Quantifying the availability of seasonal surface water and identifying the drivers of change within tropical forests in Cambodia
Source: PLoS One. 2024 Jul 29;19(7):e0307964. doi: 10.1371/journal.pone.0307964 (PMC11285917; doi:10.1371/journal.pone.0307964)
Supplement: S6 Table — Table to show the results from the Mann-Whitney U test comparing the distance to surface water from nest and non-nest points. We tested this relationship during both the dry and wet seasons. In both seasons nest points were found significantly closer to areas of surface water than non-nest points. (DOCX) [file pone.0307964.s011.docx]

**S11 Table. Table of results for comparison of distance from nest and non-nest points to surface water.**

Table to show the results from the Mann-Whitney U test comparing the distance to surface water from nest and non-nest points. We tested this relationship during both the dry and wet seasons. In both seasons nest points were found significantly closer to areas of surface water than non-nest points.

|  | **W** | **N** | **p-value** |
| --- | --- | --- | --- |
| Wet season | 221225 | 1314 | 5.52e-09 |
| Dry season | 237358 | 1314 | 2.204e-12 |
