# Supplementary material for: Quantifying the availability of seasonal surface water and identifying the drivers of change within tropical forests in Cambodia
Source: PLoS One. 2024 Jul 29;19(7):e0307964. doi: 10.1371/journal.pone.0307964 (PMC11285917; doi:10.1371/journal.pone.0307964)
Supplement: Attachment — Submitted filename: Response to Reviewers-resubmission.docx [file pone.0307964.s012.docx]

Dear Dr. Kozhikkodan Veettil,

Many thanks for your response to the submission of the paper titled ‘Quantifying the availability of seasonal surface water and identifying the drivers of change within tropical forests in Cambodia’, submission number: PONE-D-24-10904. I really appreciate you and the reviewers taking the time to evaluate this research and provide some useful feedback to improve it.

Please find a table below with information on how we have addressed and responded to each comment within the review.

| **Comment** | **Response** |
| --- | --- |
| **Editor comments:**  1.Please ensure that your manuscript meets PLOS ONE's style requirements, including those for file naming. | Completed: I have referred to the PLOS ONE formatting guidelines and have edited to my manuscript accordingly. |
| 2. In your Methods section, please provide additional information regarding the permits you obtained for the work. Please ensure you have included the full name of the authority that approved the field site access and, if no permits were required, a brief statement explaining why. | Completed: There is no specific permit number for the data collection associated with this study. I have therefore included a statement to explain this within the acknowledgements section. |
| 3. Please state what role the funders took in the study. If the funders had no role, please state: "The funders had no role in study design, data collection and analysis, decision to publish, or preparation of the manuscript." If this statement is not correct you must amend it as needed.  Please include this amended Role of Funder statement in your cover letter; we will change the online submission form on your behalf. | Completed: I have added in the sentence regarding funder involvement to my revised cover letter. |
| 4. Please note that funding information should not appear in the Acknowledgments section or other areas of your manuscript. We will only publish funding information present in the Funding Statement section of the online submission form.  Please remove any funding-related text from the manuscript. | Completed: I have removed any reference to the funders from my acknowledgements section. |
| 5. We note that you have indicated that there are restrictions to data sharing for this study. PLOS only allows data to be available upon request if there are legal or ethical restrictions on sharing data publicly. | Completed: I have added information to the data availability statement explaining why we are unable to share the giant ibis nest location data for ethical reasons. |
| 6. When completing the data availability statement of the submission form, you indicated that you will make your data available on acceptance. We strongly recommend all authors decide on a data sharing plan before acceptance, as the process can be lengthy and hold up publication timelines. Please note that, though access restrictions are acceptable now, your entire data will need to be made freely accessible if your manuscript is accepted for publication. This policy applies to all data except where public deposition would breach compliance with the protocol approved by your research ethics board. If you are unable to adhere to our open data policy, please kindly revise your statement to explain your reasoning and we will seek the editor's input on an exemption. Please be assured that, once you have provided your new statement, the assessment of your exemption will not hold up the peer review process. | Completed: I have created a github repository where all the scripts are saved and available for this project.  Completed: I have written up the Google Earth Engine scripts as .txt files so that they are accessible to anyone to read via the github repository.  Completed: I have saved and commented on the relevant R scripts for reference. These are also available via the github repository.  Completed: I have updated the access to the colab analysis script to ‘share with anyone with the link’. I have also updated the comments on this script as well. This remains available through the url in the data availability statement. |
| 7. We note that Figures 1, 3 and Supporting Figure (S6 Fig) in your submission contain map images which may be copyrighted. All PLOS content is published under the Creative Commons Attribution License (CC BY 4.0), which means that the manuscript, images, and Supporting Information files will be freely available online, and any third party is permitted to access, download, copy, distribute, and use these materials in any way, even commercially, with proper attribution. For these reasons, we cannot publish previously copyrighted maps or satellite images created using proprietary data, such as Google software (Google Maps, Street View, and Earth).  We require you to either (1) present written permission from the copyright holder to publish these figures specifically under the CC BY 4.0 license, or (2) remove the figures from your submission. | Completed: I have added in the license information into the figure captions for the data sources used to create Fig 1, Fig 3 and S6 Fig. I have attached the signed permission to publish form completed by myself and BirdLife to my resubmission. I have also added in this information to the data availability statement. I have also edited Fig 1 slightly by adding the location of Economic Land Concessions to the map. I have put it through PACE for checking and have uploaded this edited figure. |
| 8. Please include captions for your Supporting Information files at the end of your manuscript, and update any in-text citations to match accordingly. | Completed: I have added all the titles and captions of my Supporting Information at the end of the main text. I have updated these in the Supporting Information documents as well. Each Supporting Information has been saved as a separate file. |
| 9. Please review your reference list to ensure that it is complete and correct. If you have cited papers that have been retracted, please include the rationale for doing so in the manuscript text, or remove these references and replace them with relevant current references. Any changes to the reference list should be mentioned in the rebuttal letter that accompanies your revised manuscript. If you need to cite a retracted article, indicate the article’s retracted status in the References list and also include a citation and full reference for the retraction notice. | Completed: I have reviewed all my references to ensure they are all correct and included within the text. I have also converted all my references into the correct PLOS ONE format. |
| **Reviewer #1 comments**  1.Introduction:  The introduction provides a comprehensive overview of the importance of surface water and the threats it faces globally. However, it could benefit with a more detailed and articulate explanation of the study's objectives in the context of broader literature. | Completed: I have added in references to broader literature to provide more context within the introduction and to emphasise why this study’s objectives. |
| 2.Study Area:  The description of the study area is good and detailed. However, the author should provide more details about the climate of the study area, for example, different graphs and values on the average monthly and yearly rainfall, temperature, and humidity. Since rainfall is an important part of the study, different graphs of the historical average monthly, yearly, and seasonal rainfall would be beneficial. | Completed: I have added in some relevant information regarding the climate in Cambodia, specifically referencing Thoeun et al., 2015 which has detailed information on Cambodia’s climate, including a number of historical plots. |
| 3.Data Analysis:  The authors have not mentioned if any other image pre-processing is carried out besides cloud masking. It would be beneficial to mention if any other image pre-processing was carried out.  The authors should also mention why Landsat was used, instead of other sources of satellite images, like Sentient 2. | Completed: I have added in more details regarding the pre-processing of the LandSat 7 data, including more elaboration on the cloud masking and the removal of a scanline error within the data.  Completed: I have added information into the methods section explaining why we chose to LandSat 7 satellite images for this study. |
| 4.Results:  It would be recommended for the authors to carry out a trend analysis of precipitation, to see if there is any trend in precipitation if it is decreasing, and how this could be affecting the surface waters.  Additionally, it is recommended the authors provide more detail on the statistical methods used to analyze the data, including any assumptions made and how they were validated. | Completed: I have completed a linear regression to look at the trend in precipitation over time. I have added the statistical results into the results section and have added in a figure and table outlining the findings in the Supporting Information (S6 Fig and S7 Table).  Completed: I have added in more information about how the models worked, including assumptions that we made and how we validated them. |
| 5.Discussion:  It is recommended that the authors reference similar studies and make comparisons with similar studies.  Also, the authors should provide the uncertainties and limitations of the study.  Additionally, the authors should provide a conclusion section, providing a summary of key findings and including recommendations for future studies, including how the findings could inform conservation strategies or management practices. | Completed: I have added in a lot more references to similar studies and direct comparisons to this literature within the discussion.  Completed: I have included information on limitations to this study, such as not considering HEP dam influence directly within the analysis and the use of remote sensing alone with no ground truthing data.  Completed: I have added in a conclusion and recommendations section to the end of paper which summarises the key findings as well as the recommendations for future work and how the results can inform conservation strategies in this area. |
| **Reviewer #2 comments**  The general approach of the paper is very interesting and well thought. However, I would need to be able to see the code of the analysis to recommend acceptance. Without google earth engine, this seems not possible. I would therefore recommend that the authors publish the code in an open access repository. | Completed: I have cleaned and commented all of the scripts that were used for this analysis and made them available in a git hub repository. |
| **Editor comments – second round:**  We note you indicate, in your Figure captions, that some map data was derived from a source identified as "HDX".  Please: Provide a direct link to the data derived from this source, as well as the associated licensing information. | Completed: I returned to the source of the data included in this figure caption which I originally stated was from ‘HDX’ but I saw that they are originally from geoBoundaries website: <https://www.geoboundaries.org/countryDownloads.html>  I have edited the figure captions that refer to this dataset and have included the website address in the data availability statement. |
| We also note that, in your Data Availability statement, you indicate that you made extensive use of Google Earth Engine, indicating that "The study utilised existing data that are publicly available from Google Earth Engine".  Due to copyright restrictions on all Google content, PLOS is unable to publish any maps or figures which include code, basemaps, map data, etc derived from Google Earth or Google Earth Engine. Please:  -Clarify if your maps include data, code, map outlines, basemaps, etc, or any other potentially copyrighted components derived from Google Earth Engine. | Completed: the data that I am referring to in this statement is the LandSat images that I used for the analysis. I confirm that all of my figures do not contain any code, basemaps or map data derived from Google Earth or Google Earth Engine.  Figure 3 shows the results from the remote sensing analysis using the random forest model to map the change in surface water area over time. This was created using Google Earth Engine but these do not contain any data from GEE itself so I believe that there are not any copyright issues. |
| Regarding Figure 3:  -Were the LandSat images also sourced from Geoboundaries, or from a separate source? Please provide additional information regarding the source of these satellite images.  -How was Google Earth Engine used in the creation of Figure 3? If Google Earth Engine was used merely as external reference while constructing maps, and not incorporated into the map creation process, then that is acceptable. | LandSat images were sourced from the Earth Engine Data catalogue. The terms of use for the data are here:  <https://developers.google.com/earth-engine/datasets/catalog/LANDSAT_LE07_C02_T1_L2#terms-of-use>  and state:   - Landsat datasets are federally created data and therefore reside in the public domain and may be used, transferred, or reproduced without copyright restriction. - Acknowledgement or credit of the USGS as data source should be provided by including a line of text citation such as the example shown below. - (Product, Image, Photograph, or Dataset Name) courtesy of the U.S. Geological Survey - Example: Landsat-7 image courtesy of the U.S. Geological Survey - See the [USGS Visual Identity System Guidance](https://www.usgs.gov/information-policies-and-instructions/usgs-visual-identity-system) for further details on proper citation and acknowledgement of USGS products.   This is how I have cited LandSat in the paper.  As well as accessing public domain data through the catalogue, Earth Engine was used to run Javascript code written by me. All the code and results of analyses run on earth Engine are unequivocally mine. The Earth engine terms of reference are clear from the GEE website:  Who owns the algorithms I write in Earth Engine and the results of my analyses? You do. The results of all analyses you perform are yours. All algorithms that you write with our API are yours. Here's the [fine print](https://earthengine.google.com/terms/)  I hope this is sufficient to confirm there are no copyrighted data in Figure 3. |

I hope this information is enough to address the changes suggested for this manuscript. Should you require any more changes or additional information then please do not hesitate to contact me.

Sincerely

Louisa Mamalis

PhD researcher

Department of Biology

University of York

York
